# Supplementary material for: Are we throwing away good data? Evaluation of chimera detection algorithms on long-read amplicons reveals high false-positive rates across algorithms
Source: PeerJ. 2025 Dec 5;13:e20456. doi: 10.7717/peerj.20456 (PMC12684403; doi:10.7717/peerj.20456)
Supplement: Supplemental Information 1 [file peerj-13-20456-s001.docx]

**Supplementary file**


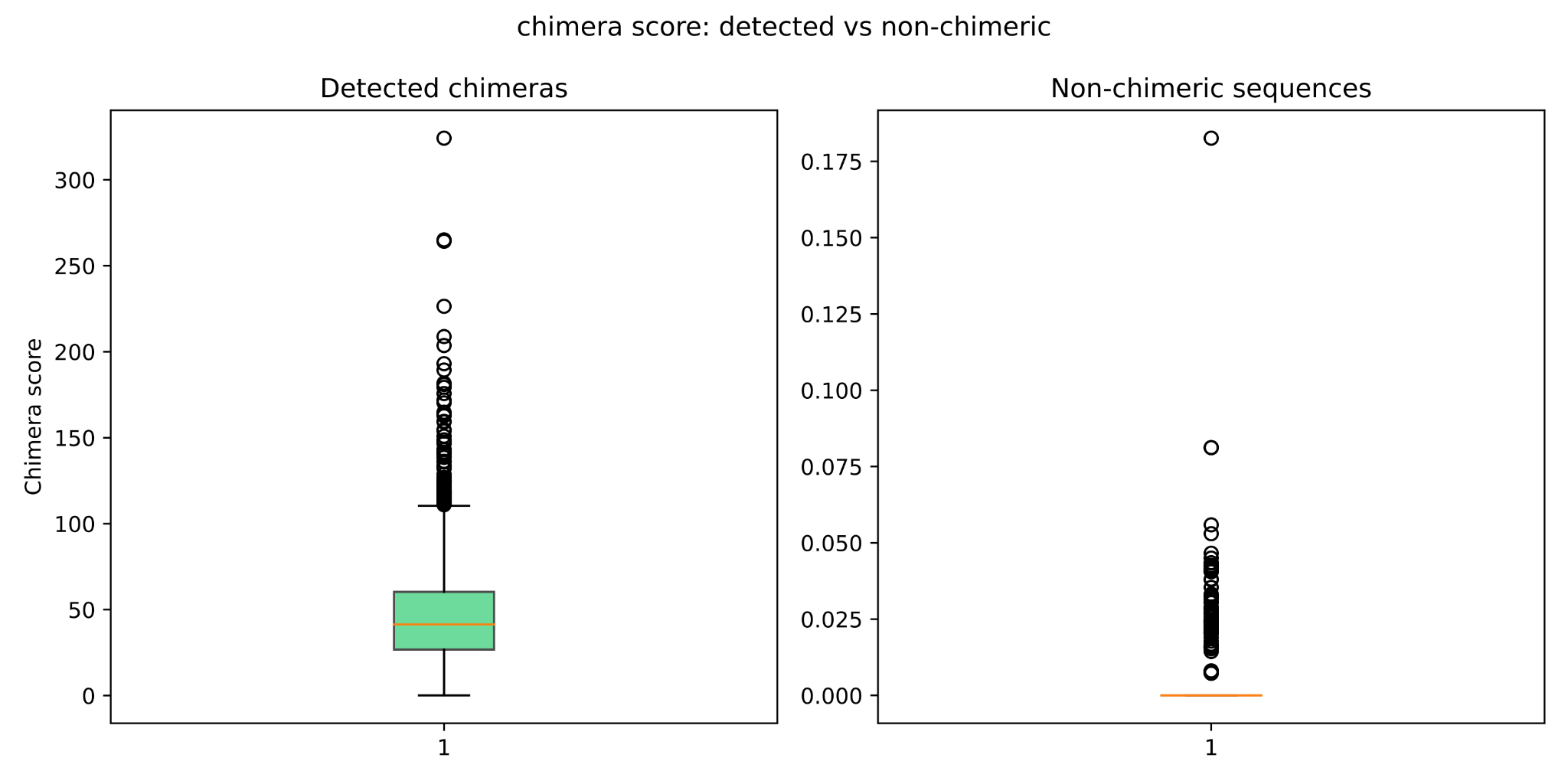


**Figure S1.** Comparison of chimera detection score distributions between *uchime_denovo* flagged chimeric sequences and verified non-chimeric sequences in the simulated dataset. The detected chimeras are related to our adjusted settings (*abskew* 3, *minh* 0.09). Since *minh*'s option determines the minimum threshold for considering a sequence as a chimera, the undetected ones obviously had lower values than 0.09 in our case.


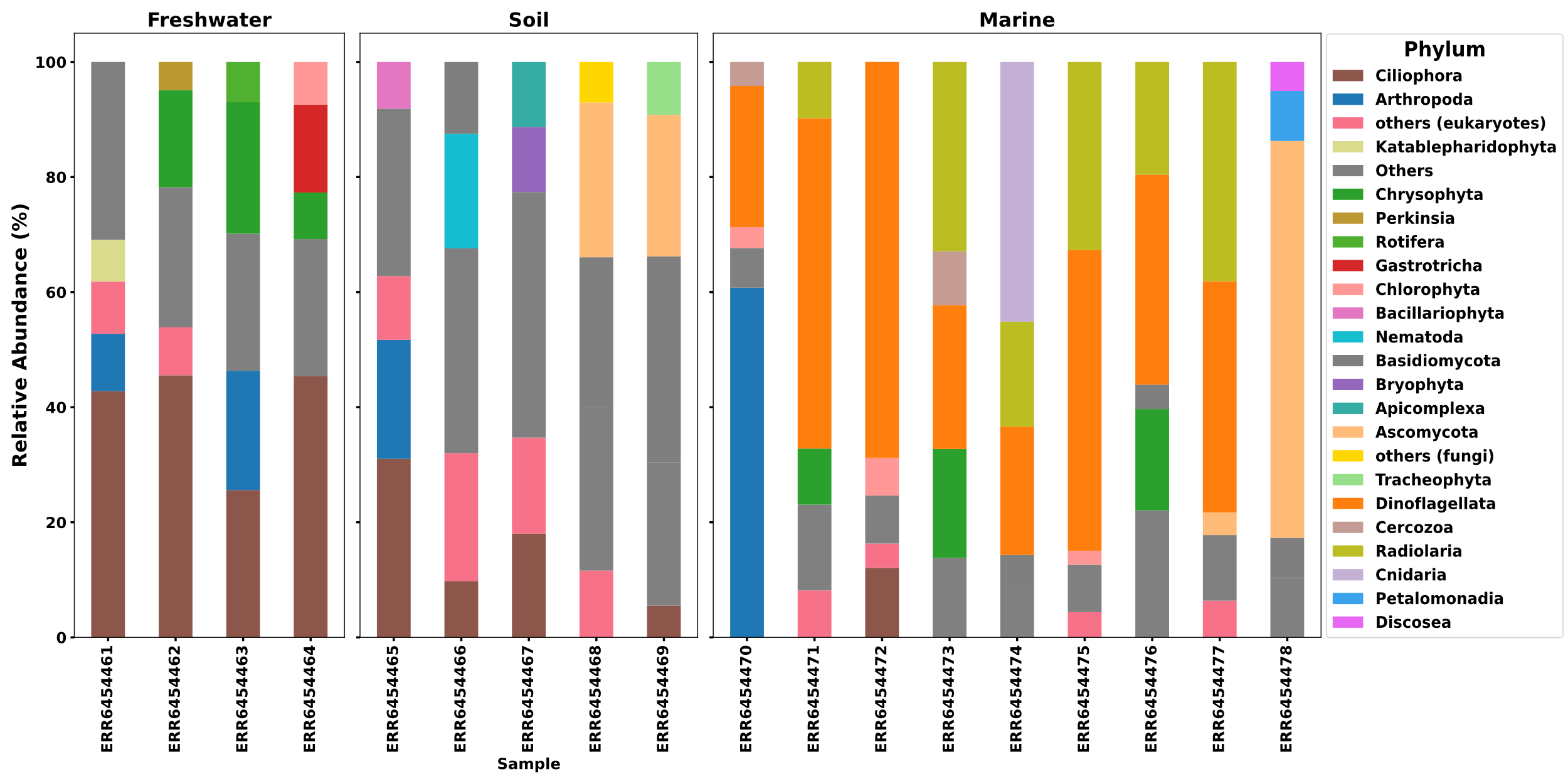


**Figure S2.** The abundance of phyla in the "non-chimeric" batch of *uchime_denovo* by adjusted settings and validated by the BLASTn. The samples related to each sample type are presented in separate boxes.


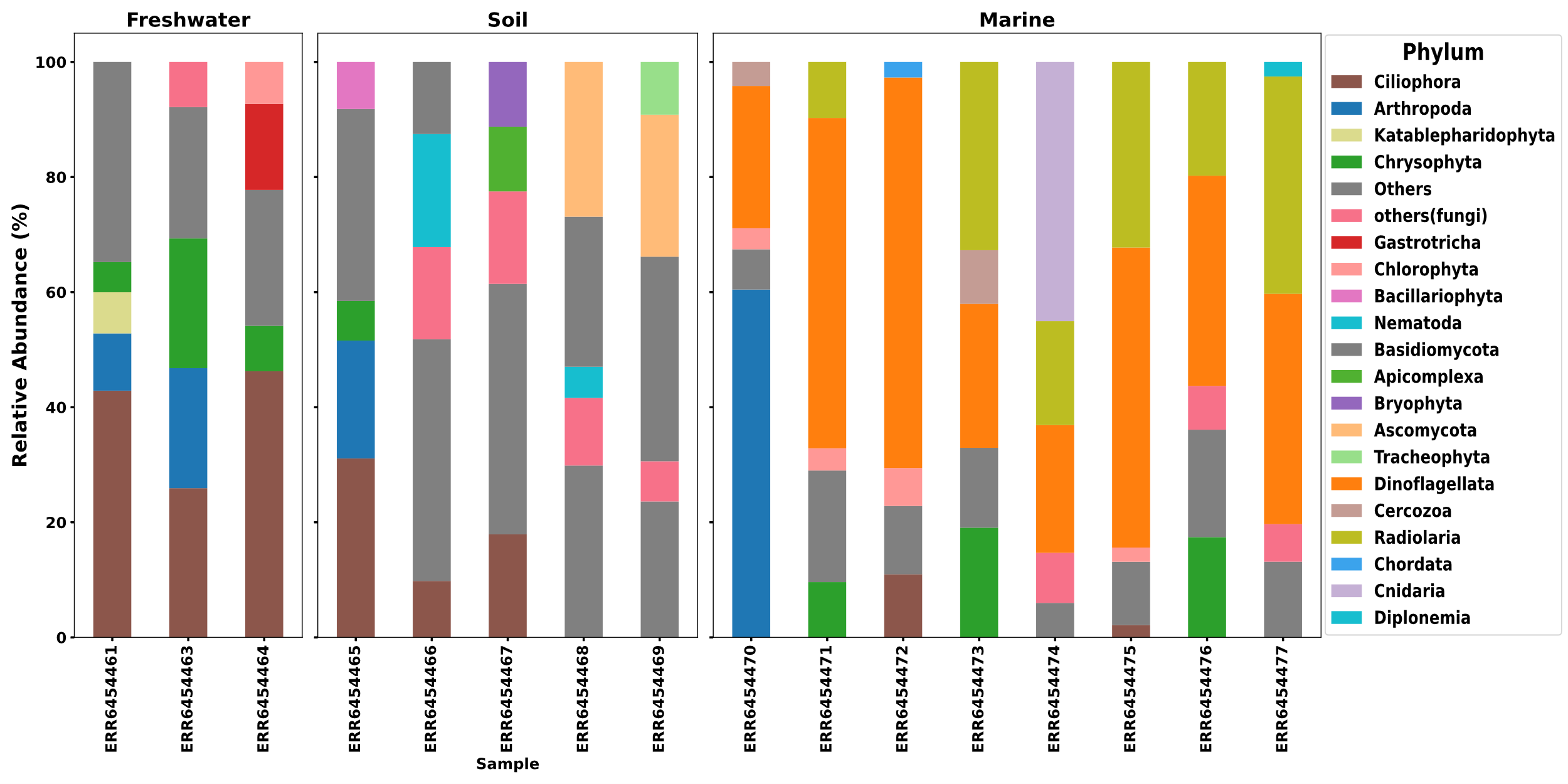


**Figure S3.** The abundance of phyla in the "non-chimeric" batch of *chimeras_denovo* was determined by adjusted settings and validated by the BLASTn. The samples related to each sample type are presented in separate boxes.

**Figure S4.** The abundance of phyla in the "non-chimeric" batch of *removeBimeraDenovo* was determined by adjusted settings and validated by the BLASTn. The samples related to each sample type are presented in separate boxes.
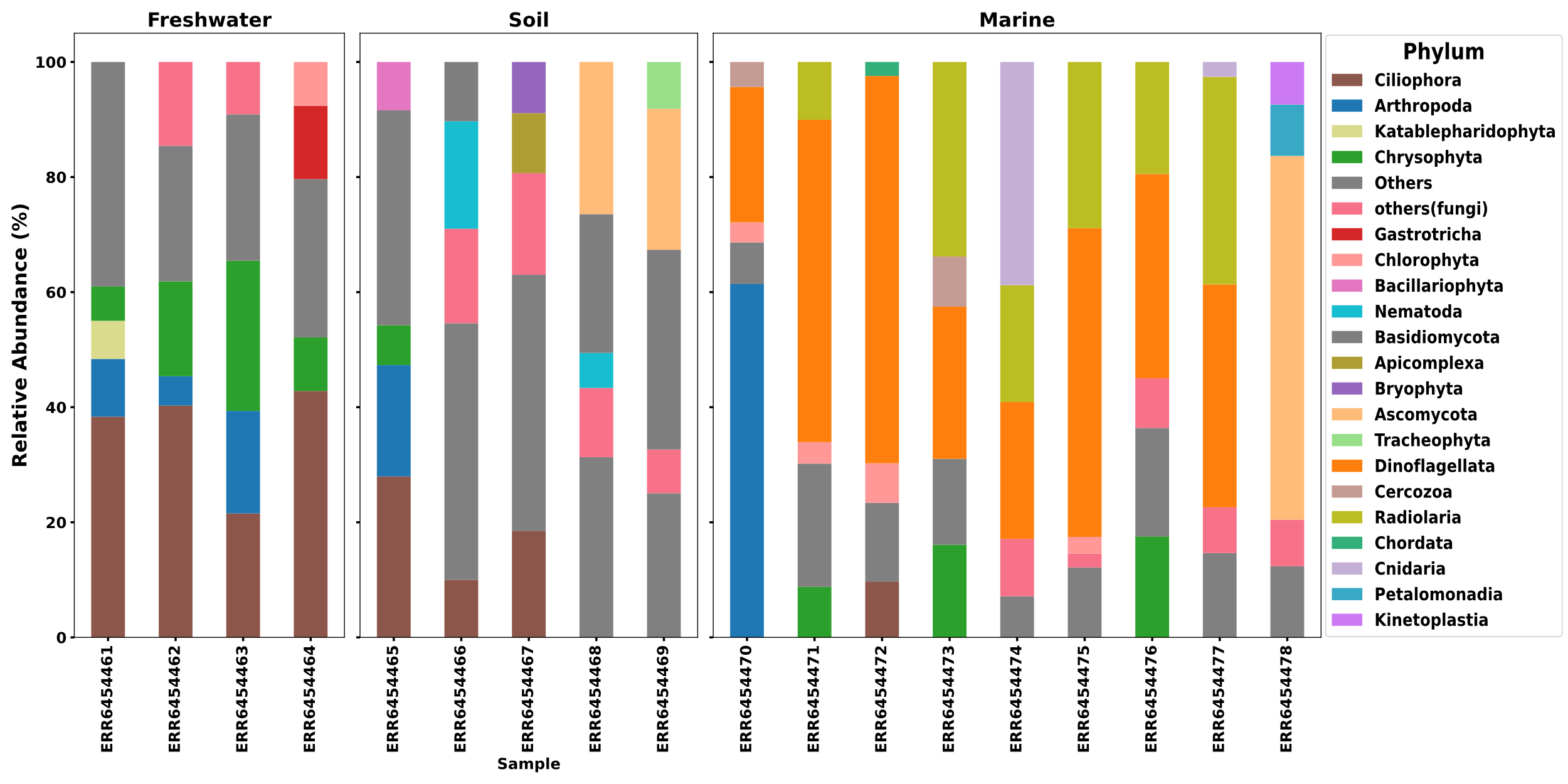


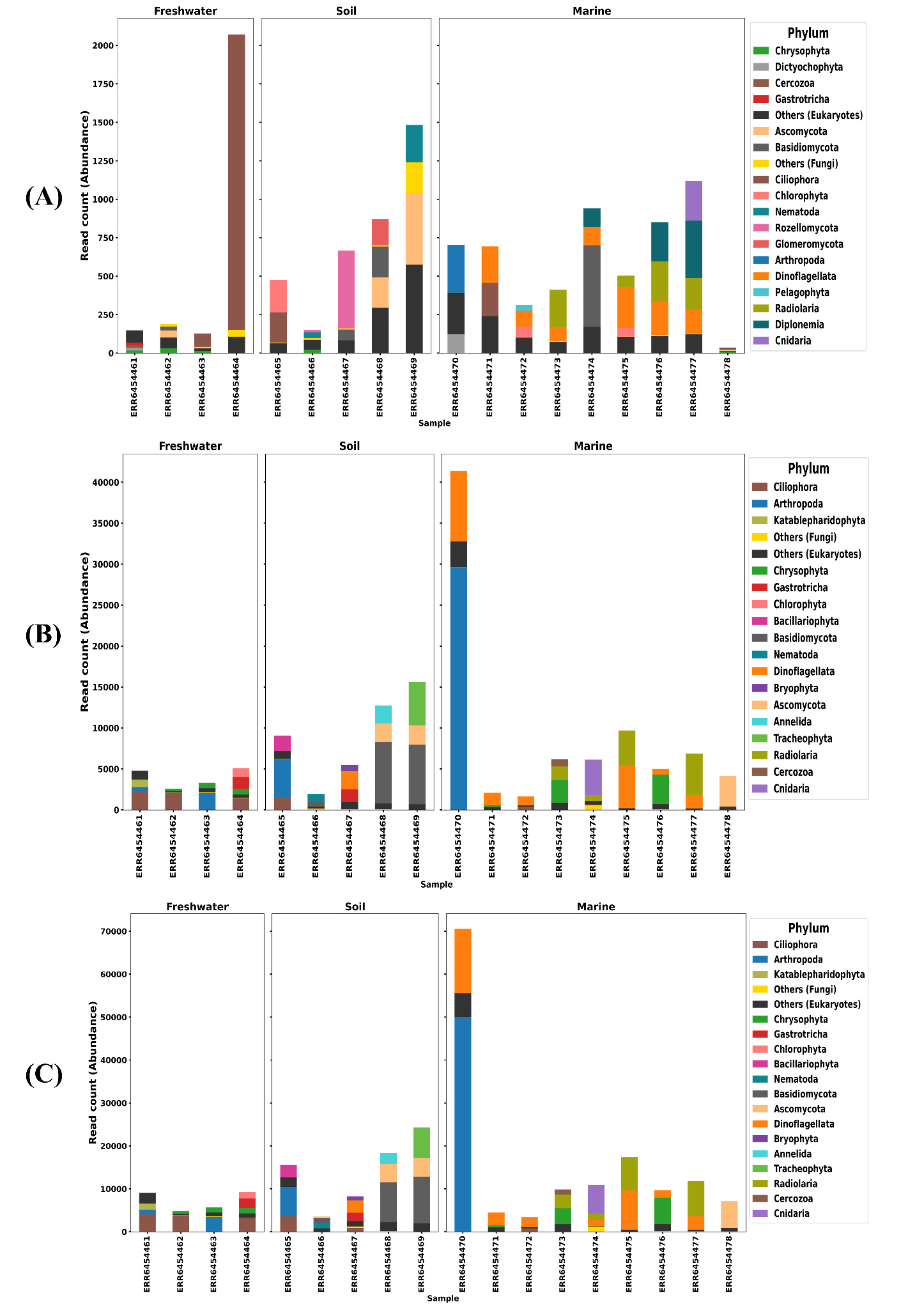
**Figure S5.** The real abundance related to phyla level of sequences in the "chimeras" batch that had a good match against the EUKARYOME database detected with the BLASTn validation module was related to each module in each sample. (A) *uchime_denovo* (B) *chimeras_Denovo* (C) *removeBimeraDenovo*. BLAST-based taxonomic assignments highlight biases in chimera detection, with fungal taxa (e.g., Basidiomycota, Ascomycota) more frequently misclassified by uchime_denovo. In contrast, non-fungal taxa (e.g., Arthropoda, Dinoflagellata) were overrepresented in chimeras_denovo false positives. DADA2 exhibited a broader taxonomic bias, impacting both fungal and non-fungal taxa.


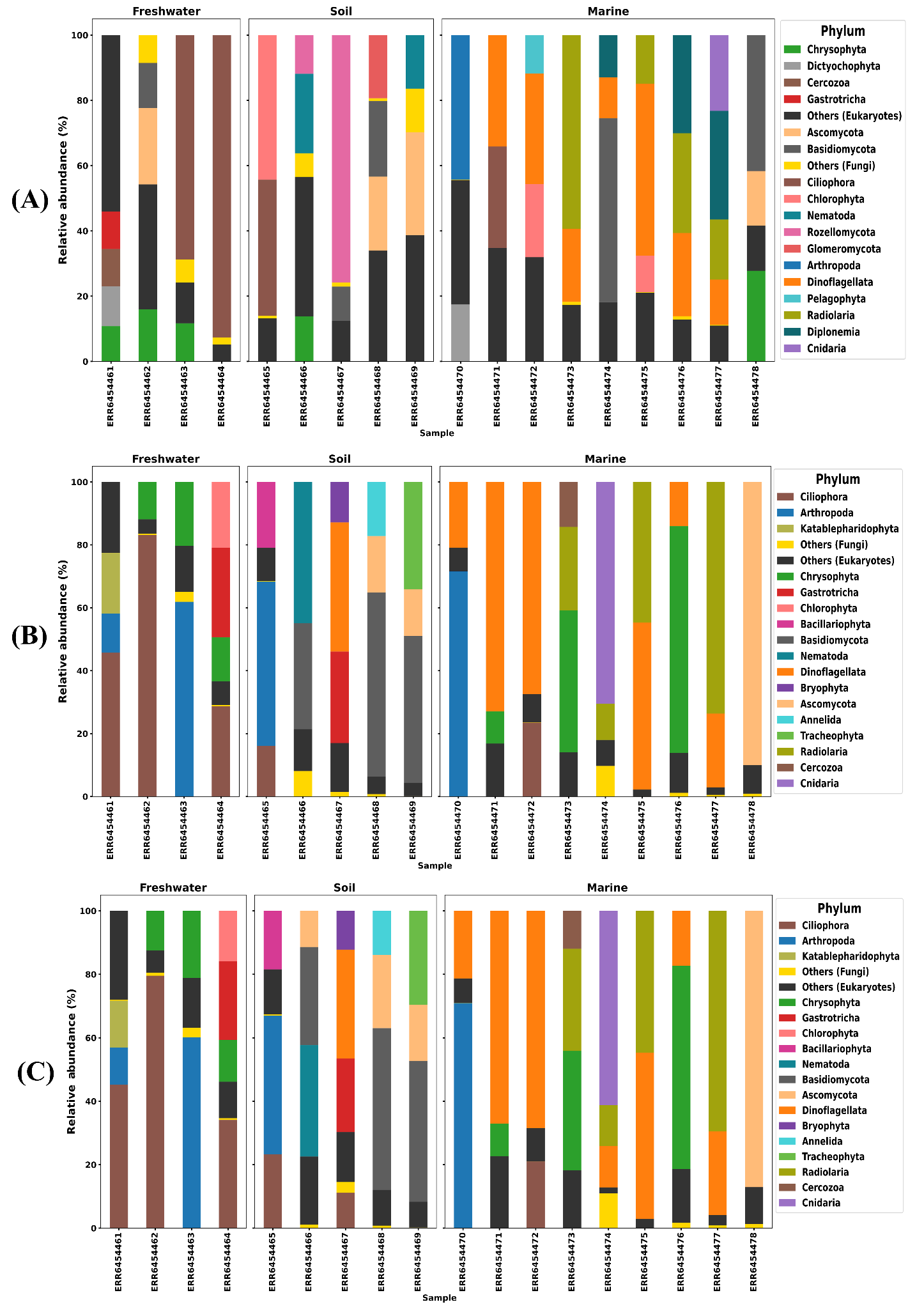


**Figure S6.** The relative abundance of phyla level sequences in the chimeras batch (after chimera filtering) that had a good match (>=99%) against the EUKARYOME database after chimera filtering step with (A) *uchime_denovo* (B) *chimeras_denovo* (C) *removeBimeraDenovo*. BLAST-based taxonomic assignments highlight biases in chimera detection, with fungal taxa (e.g., Basidiomycota, Ascomycota) more frequently misclassified by uchime_denovo. In contrast, non-fungal taxa (e.g., Arthropoda, Dinoflagellata) were overrepresented in *chimeras_denovo* false positives. *removeBimeraDenovo* demonstrates a more even distribution of misclassifications among fungal and non-fungal groups, indicating that although its taxonomic bias is wider, it is less significant for any particular taxonomic category.

**Figure S7.** The length distribution of false negative sequences in the nonchimeric batch of chimera filtering tools in the analyzed empirical dataset. The x-axis shows sequence length in base pairs, and the y-axis indicates the number of sequences recorded for each length. Blue bars show the frequency of sequences per length, and red dots mark the individual false negative sequences, indicating their specific lengths.
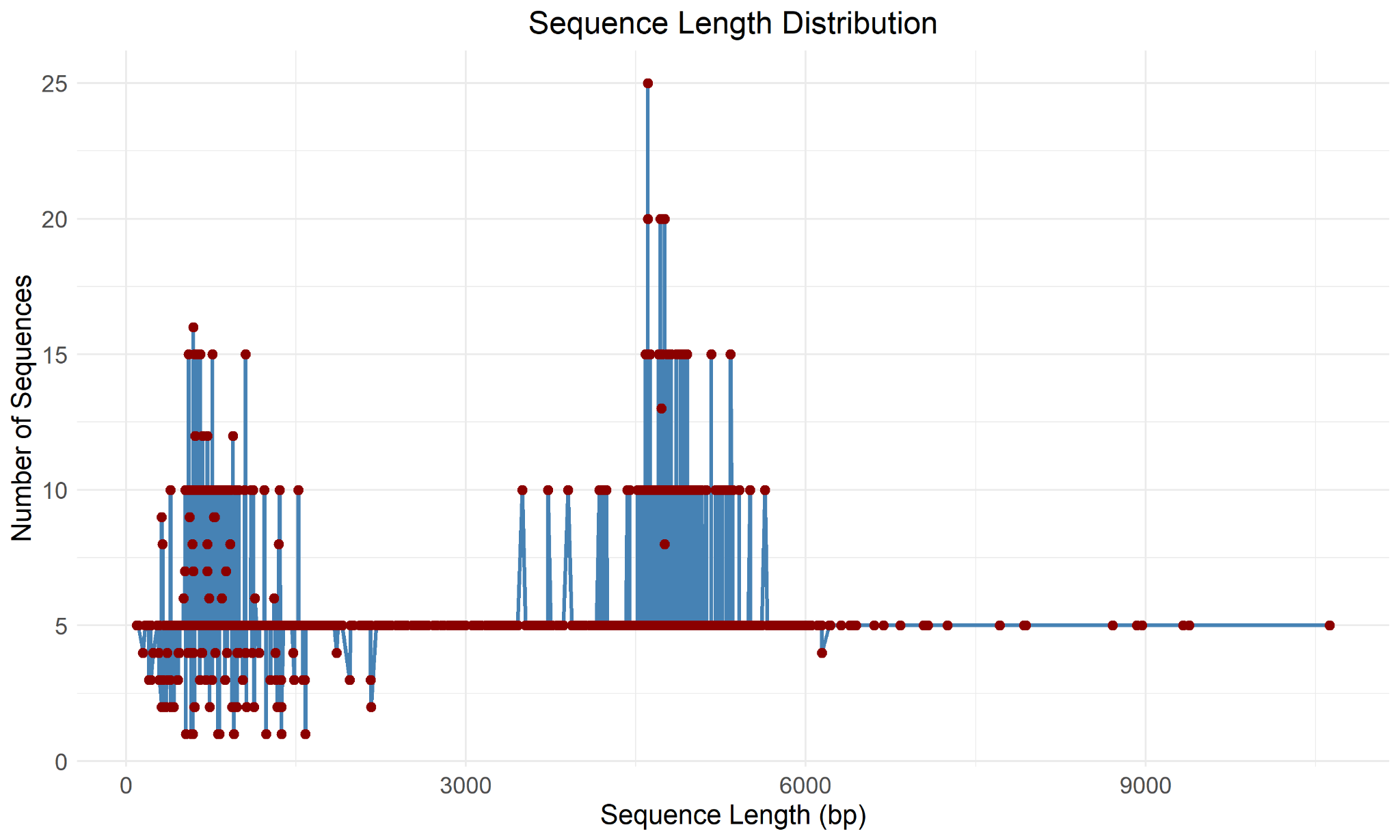


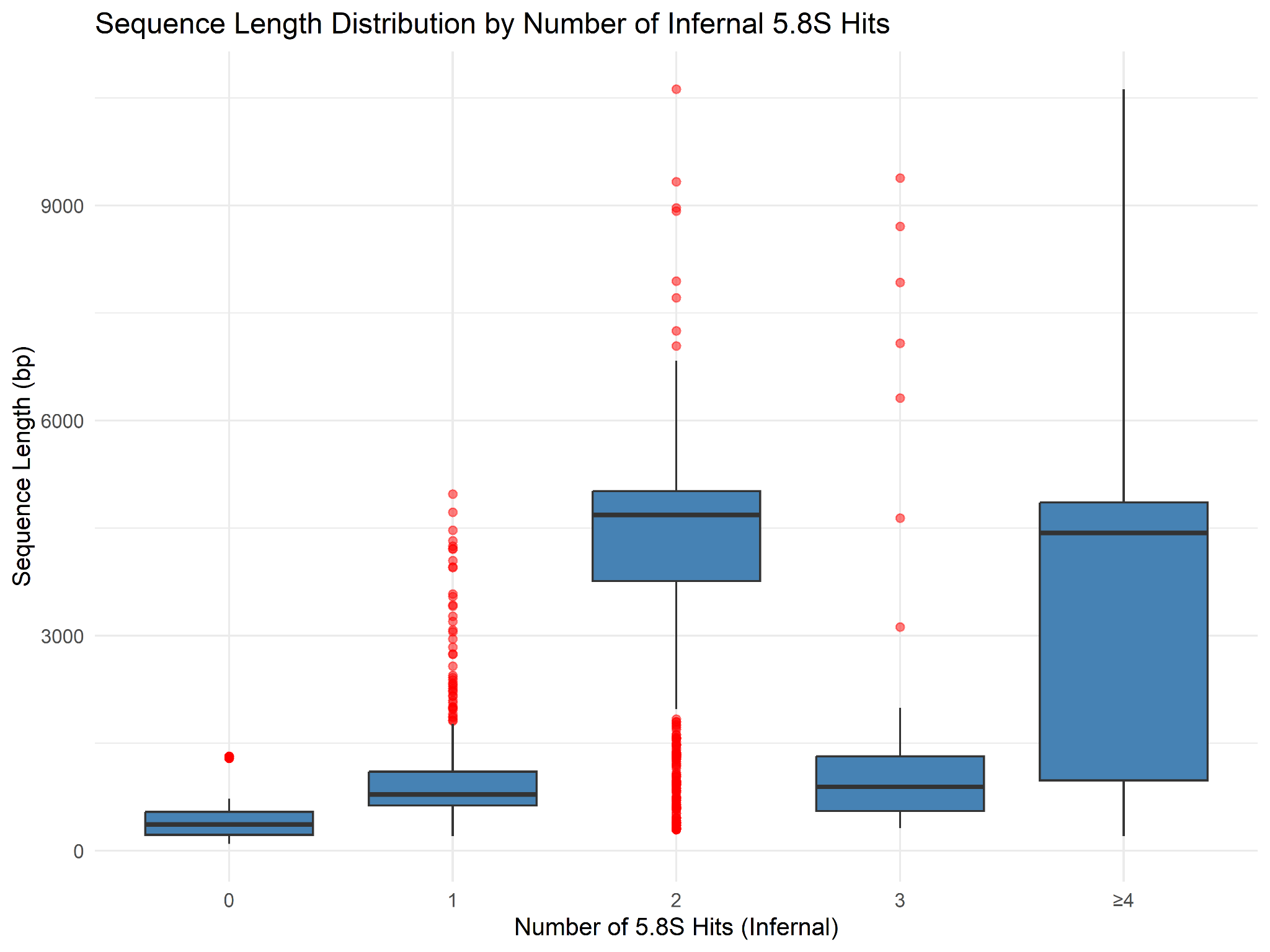
**Figure S8.** Box plot showing the distribution of all false-negative chimeric sequence lengths that remained in the nonchimeric output of algorithms related to all chimera filtering algorithms *(uchime_denovo, chimeras_denovo, removeBimeraDenovo*) grouped by the number of 5.8S hits detected using the Inferrnal package (https://github.com/brendanf/inferrnal). The x-axis ranges from zero to three hits, with an additional category for four or more (≥4). The y-axis represents sequence length in base pairs.


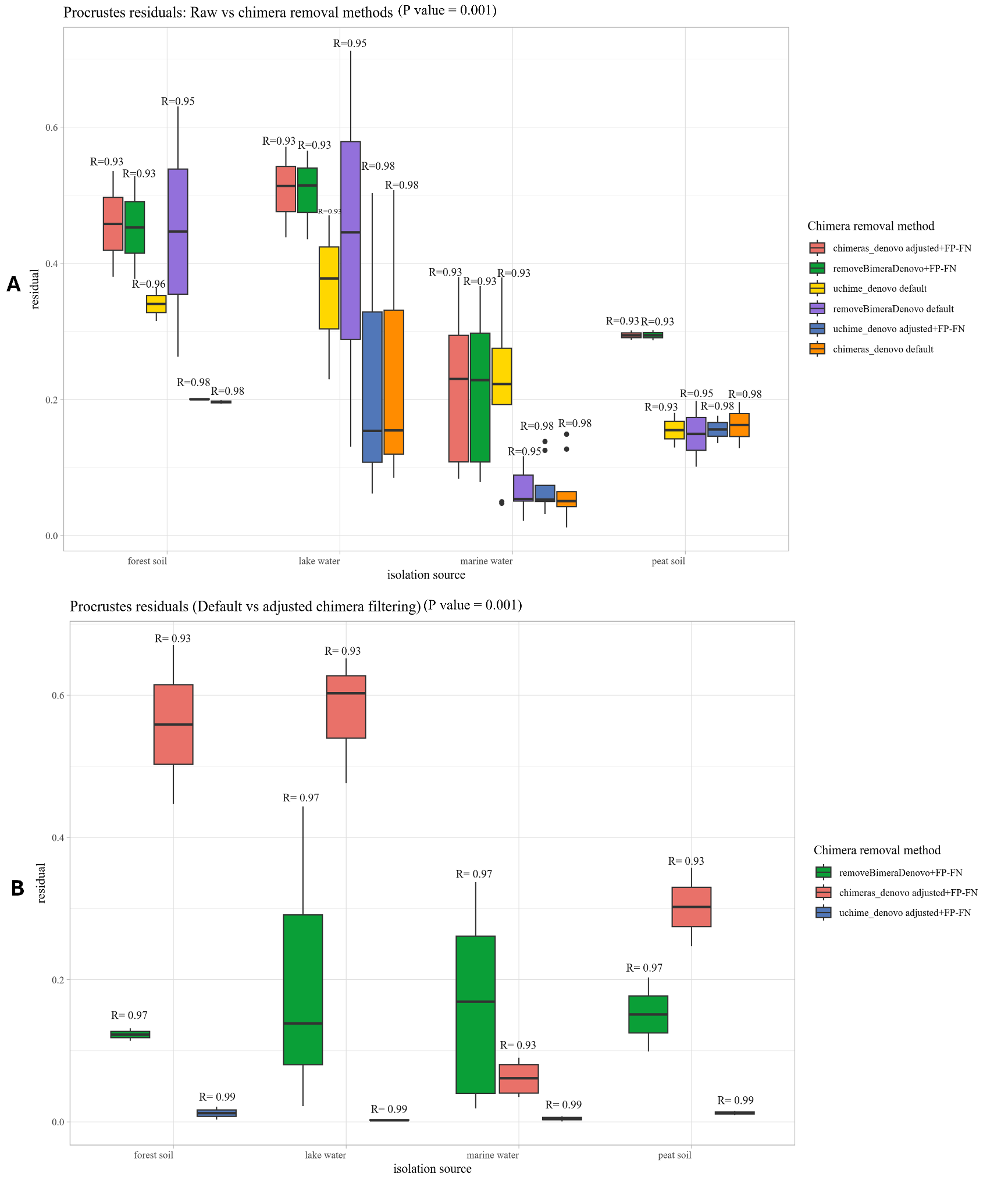


**Figure S9**. Effect of chimera-filtering algorithms on community composition across different substrates, as measured by Procrustes residuals. Boxplots display the distribution of residuals for each isolation source. For each filtering method, residuals quantify the deviation from the unfiltered data structure, with lower values indicating less disruption. Residuals were extracted for each sample; larger residuals indicate greater differences in an NMDS ordination space. R values on top of the bar represent overall Procrustes R values between compared datasets (all p = 0.001). (A) Comparison of Procrustes residuals when applying various chimera removal methods where raw OTUs data serves as a baseline. (B) Comparison of Procrustes residuals between default chimera filtered and adjusted + FP-FN OTU datasets. *adjusted+FP-FN* settings represent adjusted settings applied to each algorithm (except for *removeBimeraDenovo*), where false positives were recovered, and false negatives removed.

**Figure S10.** Comparison of Procrustes residuals between all default chimera filtering settings and all the adjusted+FP-FN approach OTU outputs for chimeras_denovo, uchime_denovo, and removeBimeraDenovo. It illustrates the impact of the adjustment and validation process on community structure representation across different isolation sources. Each panel shows the distribution of residuals calculated on NMDS ordinations of presence/absence + Bray–Curtis distances, with the Procrustes R‐value indicated in the gray strip above. Higher residuals denote greater shifts in sample relationships when false positives are rescued or false negatives removed.
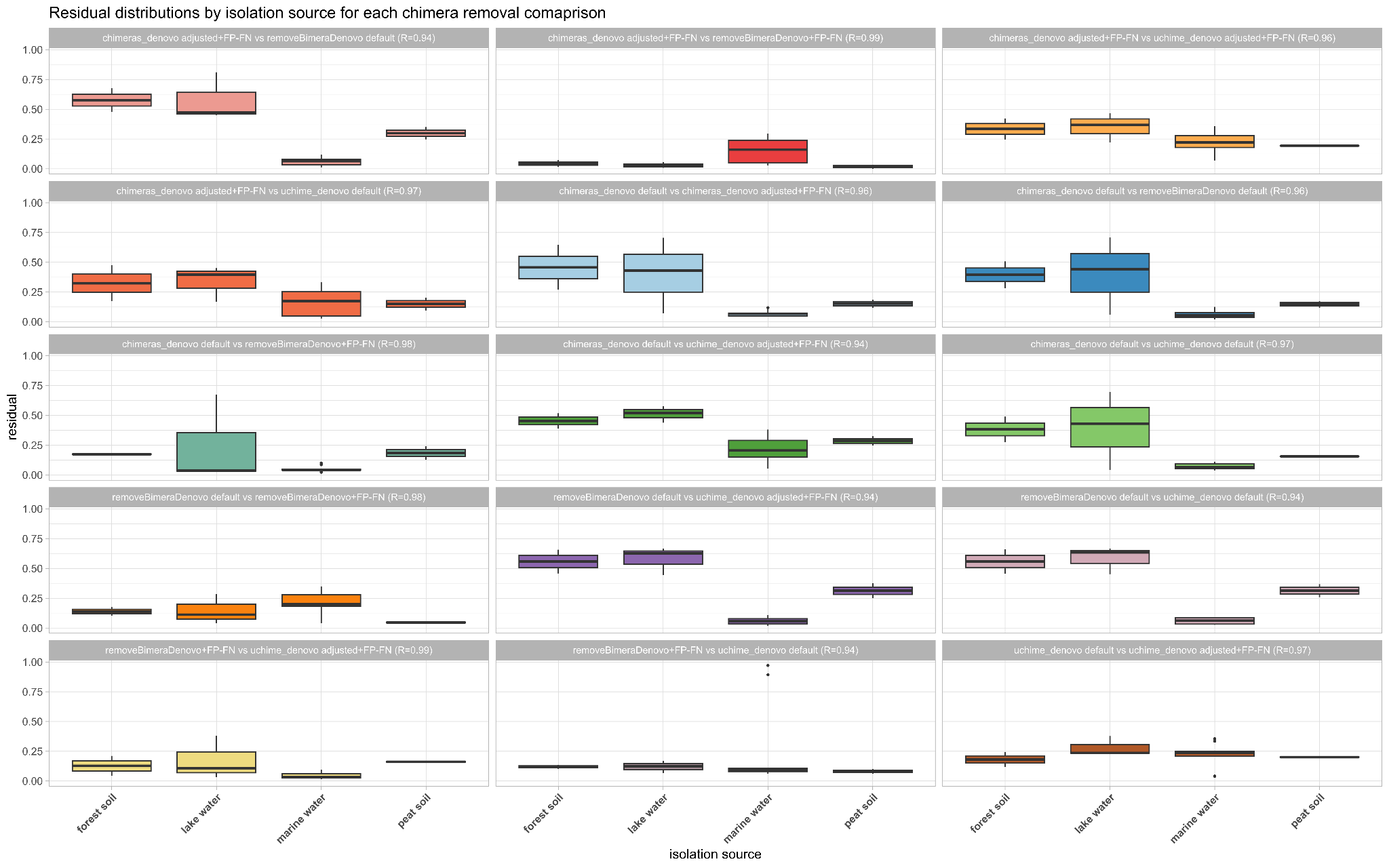


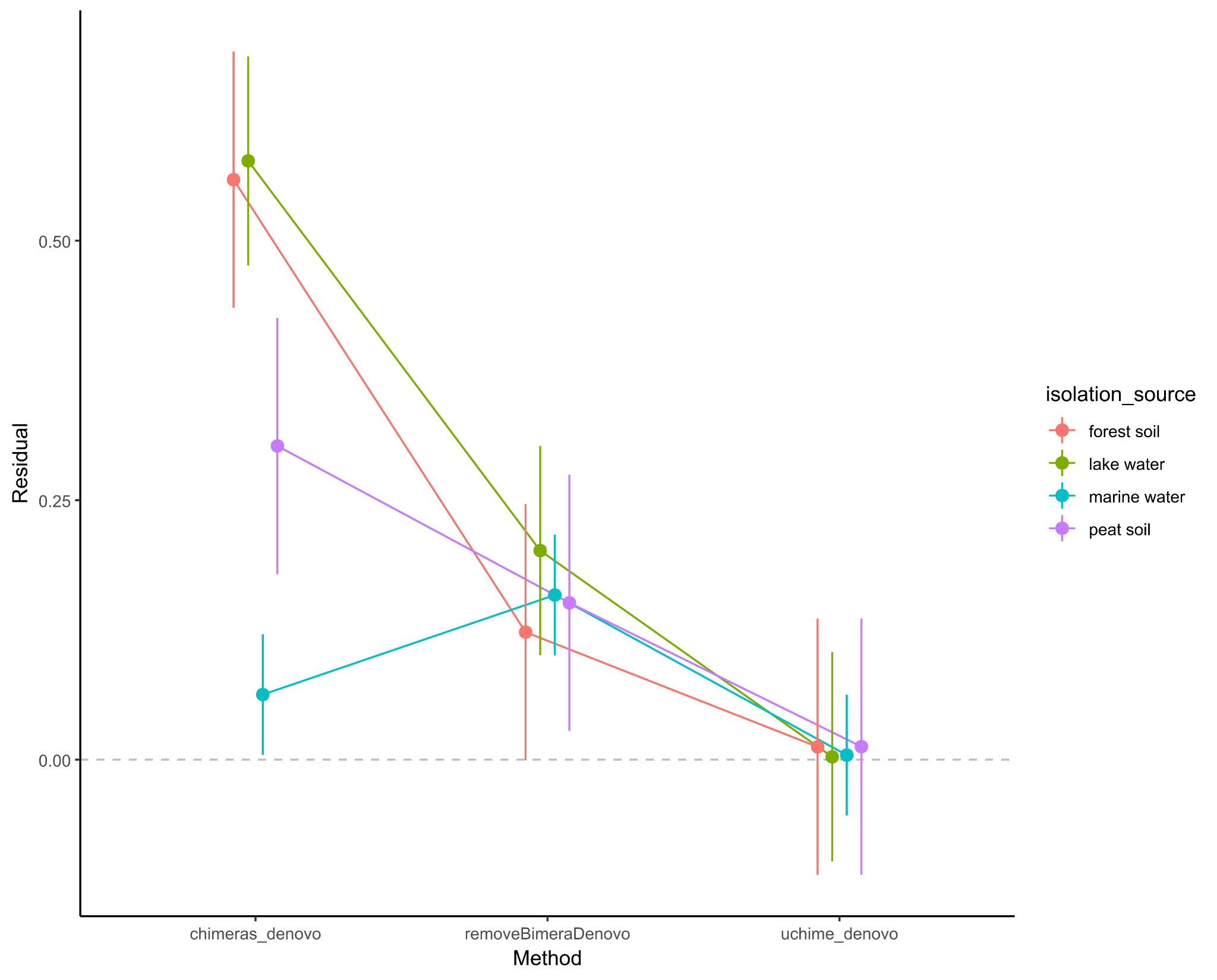


**Figure S11.** Comparison of residuals between default and adjusted chimera-filtering across methods and isolation sources. Boxplot shows the change in residuals, the per-sample deviation in NMDS ordination, when applying adjusted + FP-FN (FP-FN for *removeBimeraDenovo*) versus default parameters for chimera detection algorithms across four isolation sources. A linear mixed-effect model showed a significant method × isolation_source interaction (p<0.001), indicating that parameter adjustment alters community structure estimates in an isolation-source manner. In particular, adjusted + FP-FN causes the greatest change in *chimeras_denovo* (notably for forest soil and lake water), a moderate change in *removeBimeraDenovo*, and almost no effect on *uchime_denovo*.


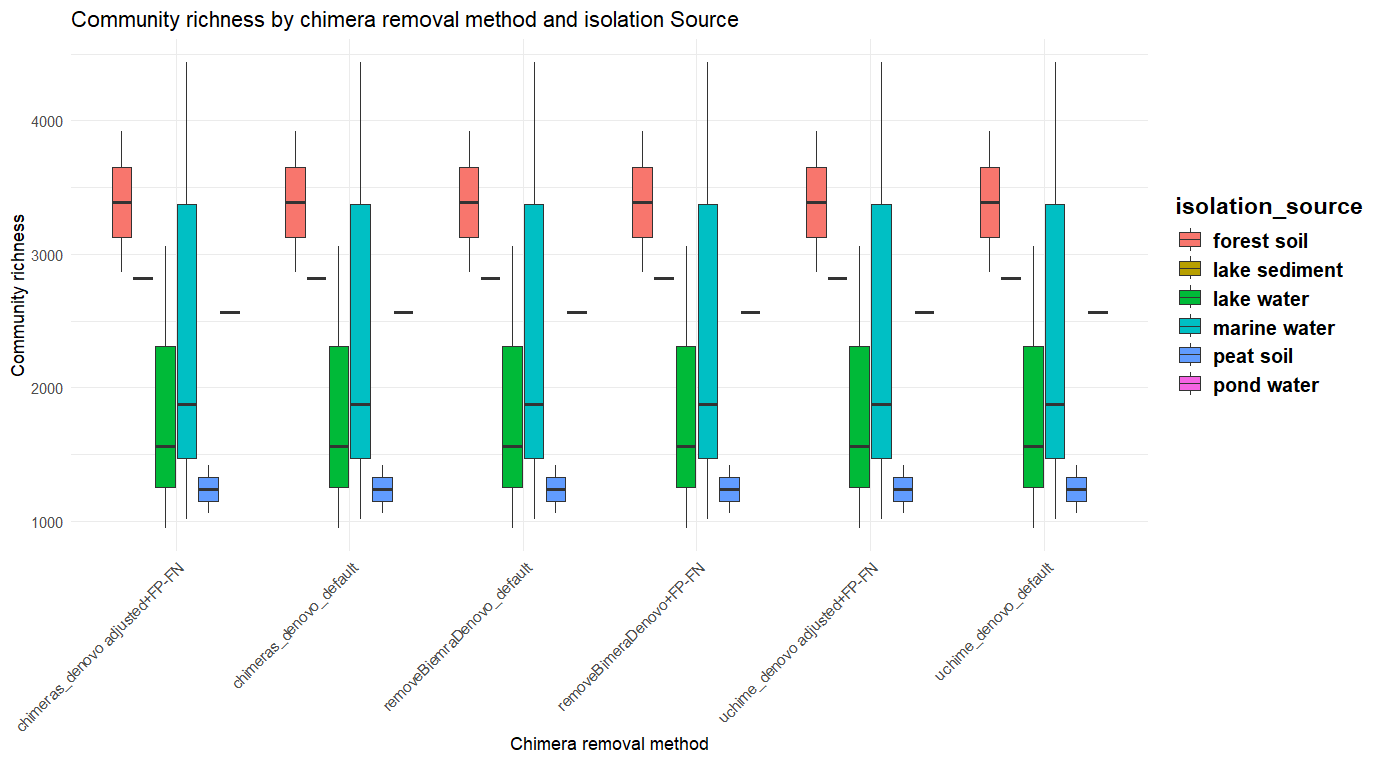


**Figure S12.** OTU richness across chimera removal methods and isolation sources. Boxplots illustrate the distribution of the richness of OTUs for each chimera removal method (x-axis) across different isolation sources. The figure demonstrates that richness varies substantially by isolation source, with forest soil samples generally exhibiting the highest richness. At the same time, the choice of the chimera removal method has little effect on the observed patterns.


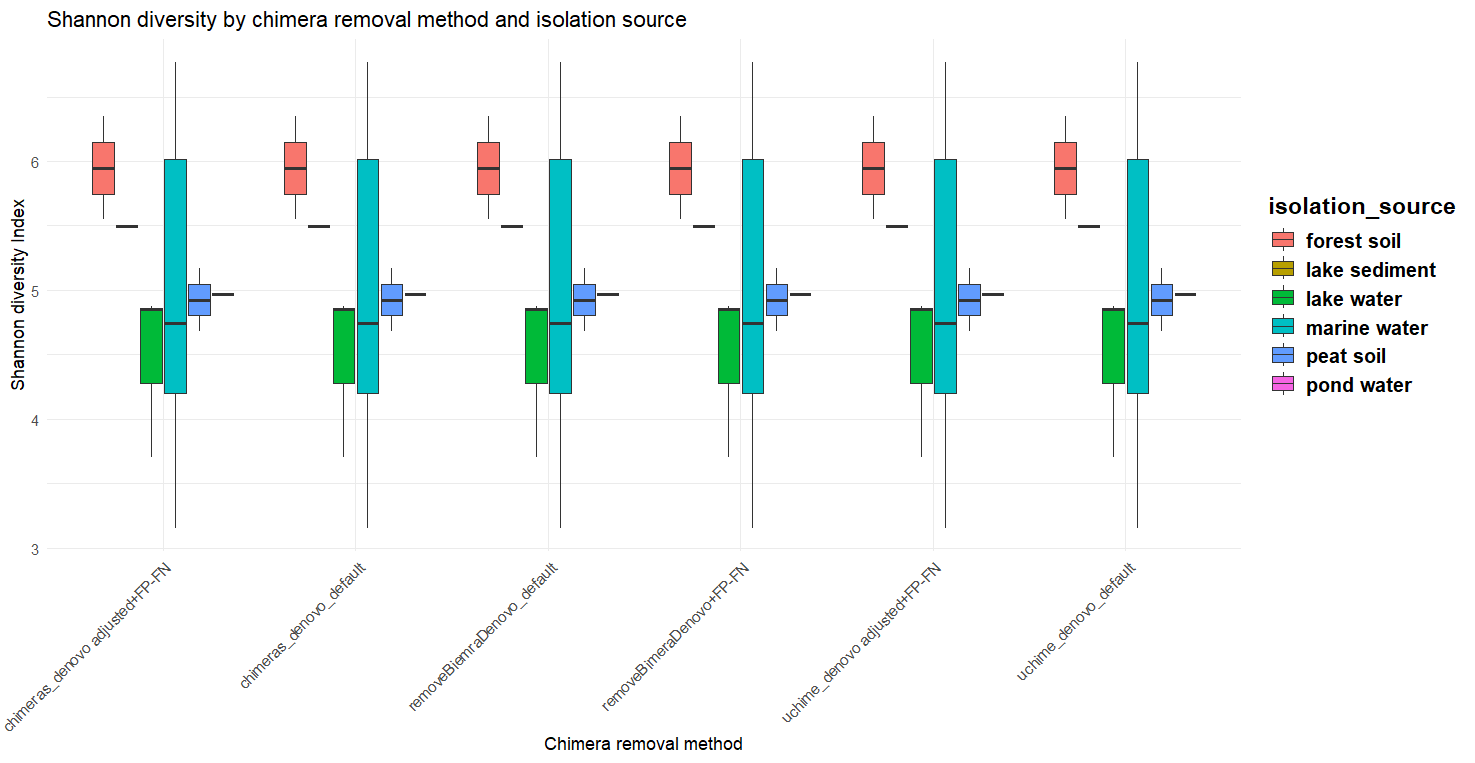


**Figure S13.** Shannon diversity index across chimera removal methods and isolation sources. Boxplots displaying the Shannon diversity index distribution for each chimera removal method (x-axis) across different isolation sources (colored boxes). The figure reveals that the isolation source strongly influences the Shannon diversity index, with forest soil samples generally exhibiting the highest diversity. At the same time, the choice of the chimera removal method shows minimal impact on the overall diversity patterns within each environment.

|  |  |  |  |  |  |  |  |
| --- | --- | --- | --- | --- | --- | --- | --- |
|  |  |  |  |  |  |  |  |
|  |  |  |  |  |  |  |  |
|  |  |  |  |  |  |  |  |
|  |  |  |  |  |  |  |  |
|  |  |  |  |  |  |  |  |
|  |  |  |  |  |  |  |  |
|  |  |  |  |  |  |  |  |
|  |  |  |  |  |  |  |  |
|  |  |  |  |  |  |  |  |
